# Heterogeneity in Category Recognition across the Visual Field

**DOI:** 10.1523/ENEURO.0331-24.2024

**Published:** 2025-01-17

**Authors:** Farideh Shakerian, Roxana Kushki, Maryam Vaziri Pashkam, Mohammad-Reza A. Dehaqani, Hossein Esteky

**Affiliations:** ^1^School of Cognitive Sciences, Institute for Research in Fundamental Sciences (IPM), Tehran 1956836613, Iran; ^2^Department of Brain and Cognitive Sciences, Cell Science Research Center, Royan Institute for Stem Cell Biology and Technology, ACECR, Tehran 141554364, Iran; ^3^Pasargad Institute for Advanced Innovative Solutions (PIAIS), Tehran 1991633357, Iran; ^4^Movement and Visual Perception Lab, Department of Psychological and Brain Sciences, University of Delaware, Newark, Delaware 19711; ^5^School of Electrical and Computer Engineering, College of Engineering, University of Tehran, Tehran 1439957131, Iran; ^6^Research Group for Brain and Cognitive Science, Shahid Beheshti Medical University, Tehran 1983969411, Iran

**Keywords:** category recognition, heterogeneity map, peripheral vision, psychophysics, visual field bias

## Abstract

Visual information emerging from the extrafoveal locations is important for visual search, saccadic eye movement control, and spatial attention allocation. Our everyday sensory experience with visual object categories varies across different parts of the visual field which may result in location-contingent variations in visual object recognition. We used a body, animal body, and chair two-forced choice object category recognition task to investigate this possibility. Animal body and chair images with various levels of visual ambiguity were presented at the fovea and different extrafoveal locations across the vertical and horizontal meridians. We found heterogeneous body and chair category recognition across the visual field. Specifically, while the recognition performance of the body and chair presented at the fovea were similar, it varied across different extrafoveal locations. The largest difference was observed when the body and chair images were presented at the lower-left and upper-right visual fields, respectively. The lower/upper visual field bias of the body/chair recognition was particularly observed in low/high stimulus visual signals. Finally, when subjects’ performances were adjusted for a potential location-contingent decision bias in category recognition by subtracting the category detection in full noise condition, location-dependent category recognition was observed only for the body category. These results suggest heterogeneous body recognition bias across the visual field potentially due to more frequent exposure of the lower visual field to body stimuli.

## Significance Statement

Our study reveals that visual object recognition exhibits notable variations across different visual field regions, with a pronounced bias in recognizing body images in the lower visual field. This heterogeneity in recognition performance suggests that the frequent exposure of certain visual field areas to specific object categories, such as bodies, influences our visual processing abilities. These findings highlight the importance of considering spatial attention and saccadic eye movements in understanding visual object recognition and have potential implications for designing more effective visual information displays and interfaces.

## Introduction

Most of the visual systems’ neural resources for shape recognition are devoted to information processing of stimuli that are projected onto the fovea. For this reason, the main focus of object vision research has been to understand the properties and neural basis of the central vision. The low resolution of stimulus representation in the peripheral visual field yields poor visual object recognition (Mäkelä et al., 2001; McKone, 2004; Jebara et al., 2009). Without saccadic eye movement, humans have the ability to instantly recognize objects utilizing their peripheral vision (Yao et al., 2011). But shape recognition for objects located in the periphery of our visual field is important in visual search and allocation of spatial attention. It plays a significant role in controlling eye movements in spatial attention and visual search (Rosenholtz et al., 2012). The peripheral visual information can improve the detection of stimuli presented at the center of gaze (Henderson and Anes, 1994) and increase the speed of reading texts (Rayner et al., 2011).

Despite the low spatial quality of peripheral vision, previous studies have reported complex object recognition of peripheral stimuli (Boucart et al., 2010) including the detection of animals in scenes (Thorpe et al., 2001; Boucart et al., 2016) and categorization of scenes (Larson and Loschky, 2009; Boucart et al., 2013; Loschky et al., 2015). Furthermore, previous studies have shown accurate recognition of faces and their emotions presented in the peripheral visual fields of humans (Hübner et al., 1985; Levy et al., 2001; Mäkelä et al., 2001; Martelli et al., 2005; Hershler et al., 2010; Bayle et al., 2011) and monkeys (Landman et al., 2014). When comparing face and house discrimination, faces were more affected than houses in parafoveal locations (Kreichman et al., 2020). Face perception is systematically influenced by the location of face presentation (Morsi et al., 2024). However, few studies have examined the recognition of body images presented at the extrafoveal visual field locations (Popivanov et al., 2016, 2015).

Our perception of visual objects across the visual field is not homogenous (Carrasco et al., 2001). Object recognition performance across the visual field decreases with eccentricity (Rijsdijk et al., 1980; Cannon, 1985; Carrasco et al., 1995; Legge et al., 2001) and at isoeccentric locations across the visual field as a function of polar angle (Carrasco et al., 2001; Fuller et al., 2008; Barbot et al., 2021). The bias in object recognition performance across the visual field depends on the task (Thomas and Nicholls, 2018; Himmelberg et al., 2020). For example, simple visual stimuli are more accurately identified at the lower compared with the upper visual field (Himmelberg et al., 2020; Barbot et al., 2021), but recognition of faces is better when presented at the upper visual field (Quek and Finkbeiner, 2016).

A bias in the representation of a particular object category, e.g., animate objects, across the vertical meridian may indicate differential processing of object category information in the two brain hemispheres. On the other hand, representational bias across the horizontal meridian may indicate experience-dependent sensory representation and/or spatial or feature attention bias across the visual field. Because predator attacks usually start from extrafoveal locations, detection of body images projected to the extrafoveal retina has survival values. Thus, peripheral body detection holds evolutionary importance in saving the prey by detecting the predator. To investigate the possibility of bias in object recognition across different parts of the visual field, we designed an experiment in which chair and animal body images with various levels of visual ambiguity were presented at the fovea and extrafoveal locations. The subjects had to decide whether the presented stimulus was a chair or an animal body. We used ambiguous stimuli to increase task difficulty. Our results show a response bias in the representation of body objects in the lower-left visual field, particularly, for the detection of noisier stimuli.

## Materials and Methods

### Participant

Sixteen healthy volunteers with normal or corrected to normal vision participated in this experiment. The study was approved in accordance with the relevant ethics committee. The test was conducted without any external interventions, such as electrical stimulation or pharmacological methods*.* The procedures and participant rights were thoroughly explained, and informed consent was obtained from all participants.

### Experiment

**Figure 1.**
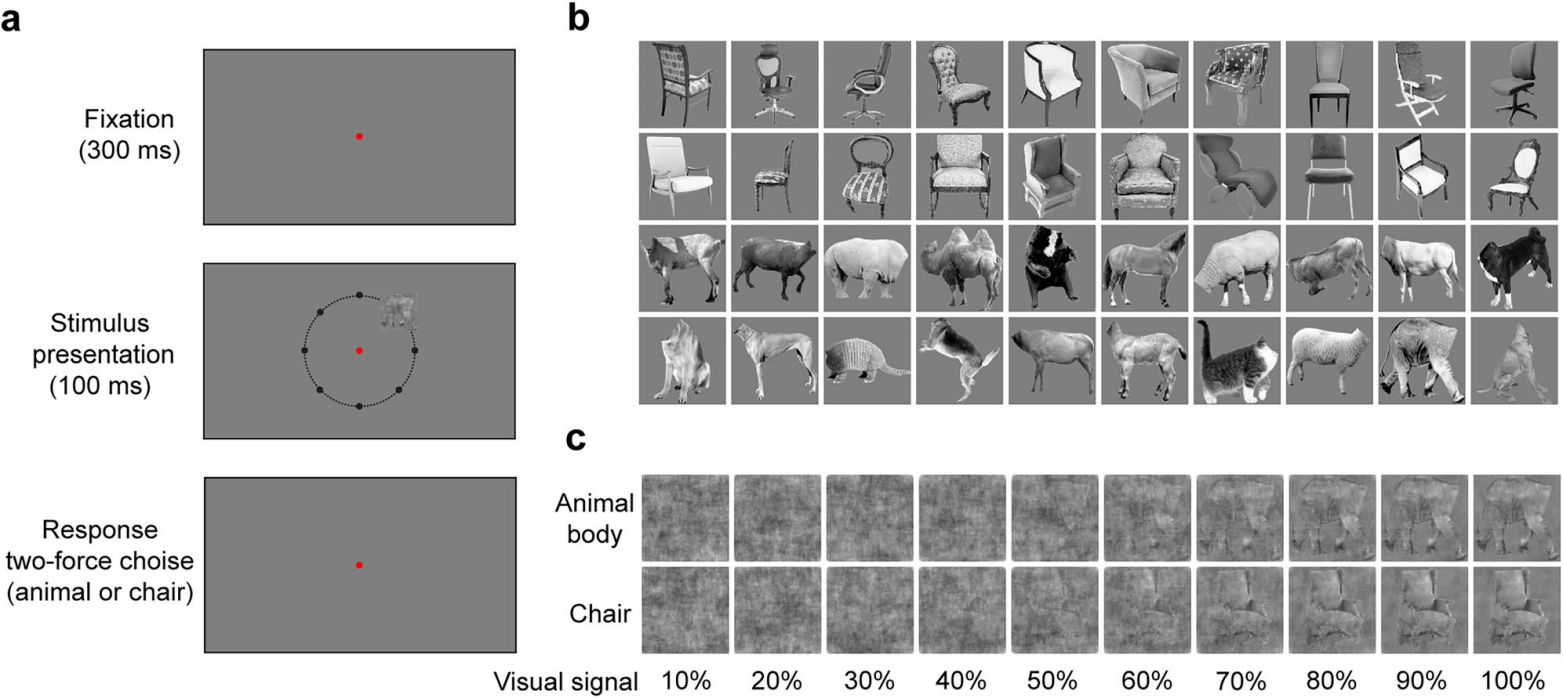
Schematic of experimental design and stimulus set. ***a***, A schematic of stimulus presentation paradigm. The stimuli were presented for 100 ms in one of the eight locations around the fixation point. The fixation point remained visible until the subject pressed a valid key. The next trial started after a 500 ms intertrial interval. A dashed line and black points show the location of the stimulus presentation for depiction purposes (note that the locations depicted in the middle plot were not shown to the subjects). They were not present in the experiment. ***b***, Chair and animal body stimuli without any noise. ***c***, Noisy stimuli of an example chair and animal body image. Numbers at the bottom indicate the levels of the visual signal.

Subjects were asked to categorize the presented stimuli as animal body or chair (Fig. 1*a*). They viewed stimuli on a 60 Hz screen located ∼57 cm from their eyes while their head rested on a chinrest in a dark room. The size of each stimulus was 2° of visual angle. The stimulus presentation was controlled using MATLAB with Psychtoolbox (Brainard and Vision, 1997) extension.

In each session, a trial started with the presentation of a red fixation point (0.3° of visual angle) in the middle of the monitor. After 300 ms fixation, a randomly selected stimulus was presented in one of eight locations at 3° of visual angle eccentricity around the fixation point for 100 ms. We asked the subjects to gaze at the fixation point during the stimulus presentation. The short stimulus presentation duration ensured that the saccade toward the stimuli could not be executed (Fischer and Ramsperger, 1984; Montagnini and Chelazzi, 2005). Subjects were instructed to choose the category of stimuli by pressing the left and right arrow keys on a computer keyboard as quickly as possible. The order of the assigned arrow keys was reversed across data collection sessions.

There were 20 animal body and 20 chair stimulus images in each signal level. Each experiment began with a training stage to familiarize the subjects with the task and stimuli. In the training phase, stimuli with different levels of the visual signal were presented at the center of the monitor. In the main experiment, 14–20 stimuli of each category with different levels of the visual signal were randomly selected and displayed once in one of eight positions (Fig. 1*a*). The number of trials across different subjects was between 1,440 to 2,240.

### Stimuli

Forty images of a four-legged animal body (from here on referred to as body) and chair stimuli were used in this experiment (Fig. 1*b*). Each category contained twenty grayscale real-world images that varied in identity, viewing angle, and pose. To create stimuli with different levels of visual ambiguity we used controlled phase randomization in Fourier space (Rainer et al., 2001). The visual signals spanned from 0% (full noise) to 100% (maximum visual signal; Fig. 1*c*). To increase the accuracy of value estimation we grouped the stimuli with different levels of visual signals into five groups: 0, 1−39, 40−59, 60−79 and 80−100%. The total luminance of stimuli was made comparable using SHINE toolbox (Willenbockel et al., 2010). All analyses were performed using MATLAB.

### Data analysis

We calculated the hit rate by assessing the subject's ability to correctly detect target stimuli (animal body or chair categories) across all trials for each level of visual signal at different locations. For each visual signal level, we measured the ratio of correct responses (i.e., trials where the subject accurately identified the target category—either body or chair) to the total number of trials presented at that signal level. Specifically, for the condition with the highest noise (i.e., the full noise level), the hit rate was calculated as the ratio of trials where the subject reported detecting the body or chair category to the total number of trials presented under this condition.

To compute the hit rate of category detection in different parts of the visual field (i.e., right, left, lower, and upper visual fields), we averaged the category hit rate for stimuli presented at each of the visual field locations. The upper/lower visual fields correspond to three locations above/below the horizontal meridian: the upper visual field includes visual angles of 45, 90, and 135°, while the lower visual field includes 225, 270, and 315°. The right/left visual fields are defined as the locations to the right/left of the vertical meridian: the right visual field includes angles of 315, 0, and 45°, while the left visual field includes 135, 180, and 225°.

To measure the presence of potential bias in subjects’ category detection across the visual field, we used an index herein called the “animacy bias index” (ABI). To calculate this index, we compared the subjects’ body and chair detection hit rate in each of the visual field locations. The ABI was defined as the normalized difference between the hit rate for body and chair detection as described below:

$$\mathrm{animacy}\;\mathrm{bia}s_{\mathrm{vsl},\mathrm{loc}}=\;\frac{{\mathrm{HR}}_{\mathrm{vsl},\mathrm{loc}}^{\mathrm{AnB}}-{\mathrm{HR}}_{\mathrm{vsl},\mathrm{loc}}^{\mathrm{Ch}}}{{\mathrm{HR}}_{\mathrm{vsl},\mathrm{loc}}^{\mathrm{AnB}}+{\mathrm{HR}}_{\mathrm{vsl},\mathrm{loc}}^{\mathrm{Ch}}},$$

where “loc” stands for location, “vsl” stands for visual signal, “AnB” represents the animal body category, “Ch” represents the chair category, and HR refers to subject’s hit rate.

In addition to the category detection, we computed the reaction time (RT) of subjects using the time of pressing arrow keys on the keyboard after stimulus offset.

We also reported *d*′ as an additional parameter to compare the subjects’ performance in detecting categories across different locations and visual signals, as described below:

$${\mathrm{d\prime}}_{\mathrm{vls},\mathrm{loc}}=\;\frac{\mu_{\mathrm{AnB}}-\mu_{\mathrm{Ch}}}{\sqrt{\frac{\left(\sigma_{\mathrm{AnB}}^{2}+\sigma_{\mathrm{Ch}}^{2}\right)}{2}}},$$

where $d_{\mathrm{vls},\mathrm{loc}}^{\prime }$ is *d*′ in each location of the visual field and visual signal, $\mu_{\mathrm{AnB}}$/ $\mu_{\mathrm{Ch}}\;$ is the average hit rate for animal body/chair, and $\sigma_{\mathrm{AnB}}^{2}$/ $\sigma_{\mathrm{Ch}}^{2}$ is the variance of animal body/chair hit rate for each location and visual signal.

### Statistical analysis

We used a two-sided Wilcoxon's signed-rank test to calculate the statistical significance in all of our analyses unless otherwise stated. We tested the distribution of hit rate differences by using the Kolmogorov–Smirnov (KS test). Analysis of variance (ANOVA) was used to examine the interaction of visual field location and the stimulus visual signal. The average values were reported as mean ± SEM. $\Delta_{\left(a,b\right)}^{c}$ was used to address a difference between the hit rate of a and b in situation c. To compare the minimum and maximum response ranges across all locations, we applied FDR correction to adjust the *p*-values, as multiple statistical tests were performed simultaneously when analyzing data from 16 subjects across eight locations. All statistical analyses were conducted using MATLAB software with the Statistical Toolbox.

### Code accessibility

The code used to create figures can be found at https://github.com/FShakerian/visual–field-heterogeneity.

## Result

The main objective of this study was to investigate a potential bias in body and chair category recognition bias across different parts of the visual field. To examine this potential bias, we designed a two-alternative force choice task in which subjects had to categorize body or chair stimuli with different levels of visual signal. The stimuli were presented in one of eight locations around the fixation point (Fig. 1).

**Figure 2.**
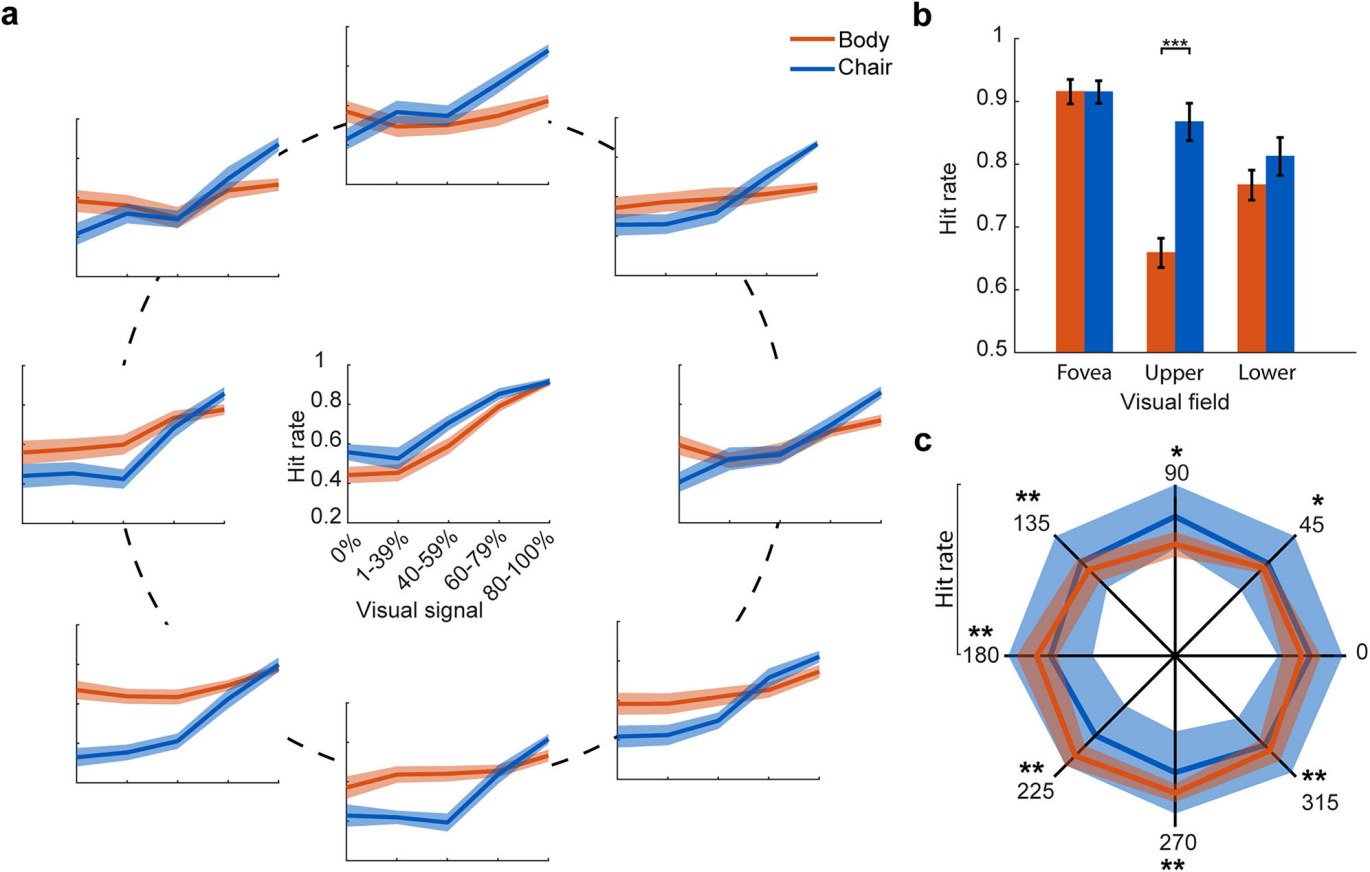
Differences in subjects’ hit rate for body and chair categories. Subjects’ performance in detecting body and chair categories varies between foveal and extrafoveal locations. ***a***, The response curve of subjects (mean ± SEM) across the visual field for different levels of visual signal. ***b***, Average hit rate across subjects for detecting body and chair at the fovea, upper (45, 90, and 135°), and lower (225, 270, and 315°) visual fields for less noisy stimuli. ***c***, Hit rate range across levels of visual signals is plotted for each location. The shaded areas show the hit rate range (max–min). *p*-values of two-sided Wilcoxon's signed-rank test are reported for each location. *p < 0.05, **p < 0.01, ***p < 10^−3^.

The response curve in Figure 2*a* showed that subjects’ performance in detecting body and chair categories differs between the foveal and extrafoveal locations. To identify these differences, we compared the subjects’ hit rate, $\Delta_{\left(\mathrm{chair},\mathrm{animal}\right)}^{\mathrm{location}},$ for less noisy stimuli (80–100% visual signal) between the foveal and extrafoveal locations. When stimuli were presented at the center of the gaze, subjects’ category detection hit rates were similar for body and chair category (Fig. 2*b*; $\Delta_{\left(\mathrm{chair},\mathrm{animal}\right)}^{\mathrm{fovea}}$ = −0.0001 ± 0.02, *p* = 0.9). However, comparing hit rate in the lower and upper extrafoveal locations showed that chair detection was more than body detection (Fig. 2*b*; $\;\Delta_{\left(\mathrm{chair},\mathrm{animal}\right)}^{\mathrm{upper}}$ = 0.23 ± 0.04, *p* < 10^−4^; $\;\Delta_{\left(\mathrm{chair},\mathrm{animal}\right)}^{\mathrm{lower}}$ = 0.06 ± 0.03, *p* = 0.09). Body detection is more sustained than chair detection across different levels of visual signals. To assess the fluctuations in body and chair detection, in Figure 2*c*, we calculated the maximum and minimum hit rates for body and chair detection across various visual signals for each subject, at each location. The shaded blue and red areas represent the range of minimum to maximum responses across subjects, while the red and blue lines show the average hit rates for body and chair detection at each location. The numerical range from minimum to maximum values is displayed in Table 1.

**Table 1.** Hit rate range (difference of minimum and maximum hit rate) in each location for body and chair category

Location	Body	Chair	*p*-value
0°	0.30 ± 0.04	0.41 ± 0.04	0.17
45°	0.28 ± 0.03	0.44 ± 0.04	0.02
90°	0.25 ± 0.02	0.37 ± 0.04	0.04
135°	0.26 ± 0.04	0.45 ± 0.03	0.01
180°	0.26 ± 0.04	0.48 ± 0.04	0.01
225°	0.25 ± 0.03	0.49 ± 0.04	0.005
270°	0.23 ± 0.02	0.46 ± 0.04	0.005
315°	0.25 ± 0.03	0.45 ± 0.04	0.003

The reported *p*-values are adjusted with FDR correction.

**Figure 3.**
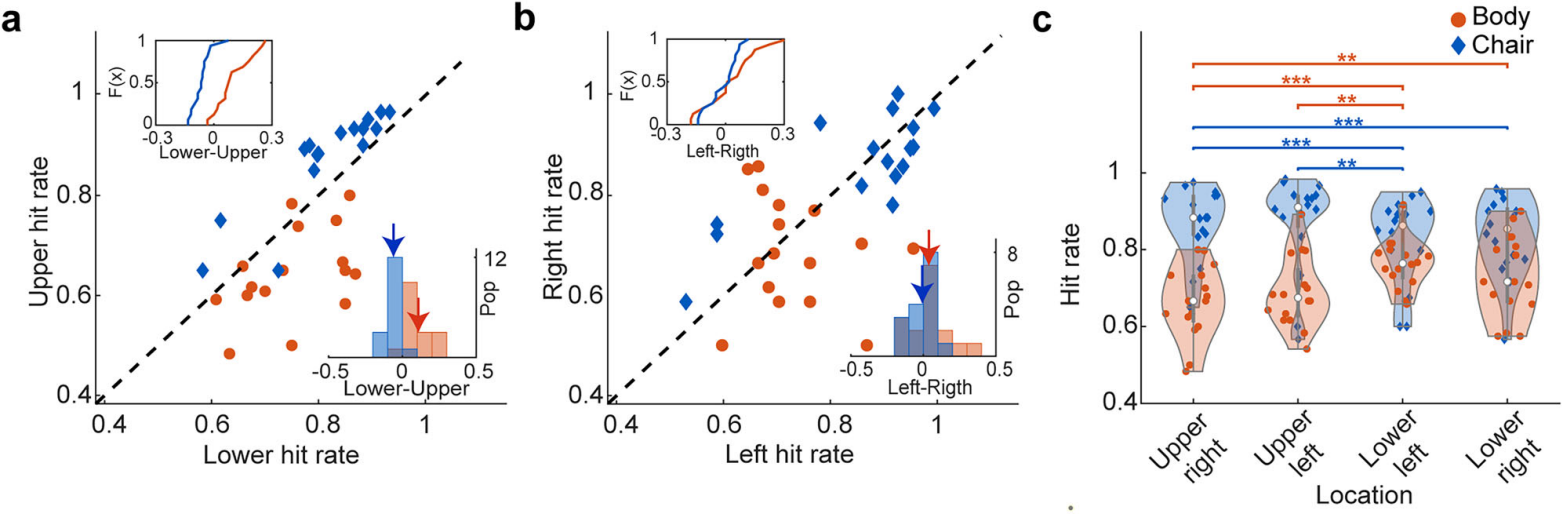
Subjects’ hit rate to detect less noisy stimuli (80–100%) of body and chair. There was variability in responses within the body and chair categories across the extrafoveal locations of the visual field. ***a***, Individual subjects’ hit rate in the lower and upper visual fields. The histogram in the bottom right corner shows the population distribution of lower minus upper visual field hit rate for chair and body. The plot in the top left part shows the cumulative distribution of the difference between lower and upper visual field hit rates. ***b***, Individual subjects’ hit rate in the right and left visual fields. The arrows show the mean of the distributions. ***c***, The violin plot shows the hit rate in each visual field quadrant. We calculated the average hit rate across three neighboring locations for each quadrant (e.g., averaging 0, 45, and 90° for the upper-right quadrant). Chair is plotted in blue and body in red; *n* = 16. *p < 0.05, **p < 0.01, ***p < 10^−3^.

In addition to the difference between the chair and body detection, there was also heterogeneity in the response within each category across the extrafoveal locations of the visual field. The hit rates of subjects in detecting body and chair images at the highest level of visual signal (80–100%) are shown in a scatter plot (Fig. 3*a*). The subjects’ hit rate to detect body was greater in the lower visual field (*p* < 10^−3^, Wilcoxon's signed-rank test), while for chair detection, it was greater in the upper visual field (*p* = 0.002, Wilcoxon's signed-rank test). There was a significant difference between the distribution of detection difference for body and chair images (empirical cumulative distribution for body and chair images; Fig. 3*a*, bottom right subpanel; $\;\;\Delta_{\left(\mathrm{lower},\mathrm{upper}\right)}^{\mathrm{animal}}$ = 0.11 ± 0.02, ${\;\Delta }_{\left(\mathrm{lower},\mathrm{upper}\right)}^{\mathrm{chair}}$ = −0.06 ± 0.01, KS test, *p* < 10^−6^). The lower field bias for body detection and the upper field bias for chair detection were observed across all levels of visual signal (Table 2).

**Table 2.** Mean hit rate of the upper and lower visual fields for body and chair detections

Visual signal	Category	Mean hit rate ± SEM		*p*-value
		Upper visual field	Lower visual field	
1–39%	Animal body	0.54 ± 0.0 4	0.61 ± 0.04	0.1
	Chair	0.48 ± 0.0 3	0.41 ± 0.04	0.002
40–59%	Animal body	0.55 ± 0.03	0.63 ± 0.03	0.01
	Chair	0.50 ± 0.03	0.43 ± 0.03	0.004
60–79%	Animal body	0.61 ± 0.04	0.68 ± 0.02	0.05
	Chair	0.71 ± 0.06	0.65 ± 0.04	0.15

The *p*-values reported compare the upper and lower hit rates for each category.

This significant bias was not in the left and right visual fields. (Fig. 3*b*, scatter plot; chair, *p* = 0.92; body, *p* = 0.13), and the hit rate difference was not statistically significant (Fig. 3*b*, the histogram on the bottom right subpanel; ${\;\;\Delta }_{\left(\mathrm{left},\mathrm{right}\right)}^{\mathrm{animal}}$ = 0.05 ± 0.03, $\Delta_{\left(\mathrm{left},\mathrm{right}\right)}^{\mathrm{chair}}$ = −0.01 ± 0.01, KS test, *p* = 0.16). As depicted in Figure 3*c*, the largest body detection was observed in lower quadrants (upper-right, 0.68 ± 0.02; upper-left, 0.71 ± 0.02; lower-left, 0.76 ± 0.01; lower-right, 0.72 ± 0.02), and the largest chair detection was observed in the upper quadrants (chair: upper-right, 0.86 ± 0.02; upper-left, 0.87 ± 0.02; lower-left, 0.83 ± 0.01; lower-right, 0.85 ± 0.02).

**Figure 4.**
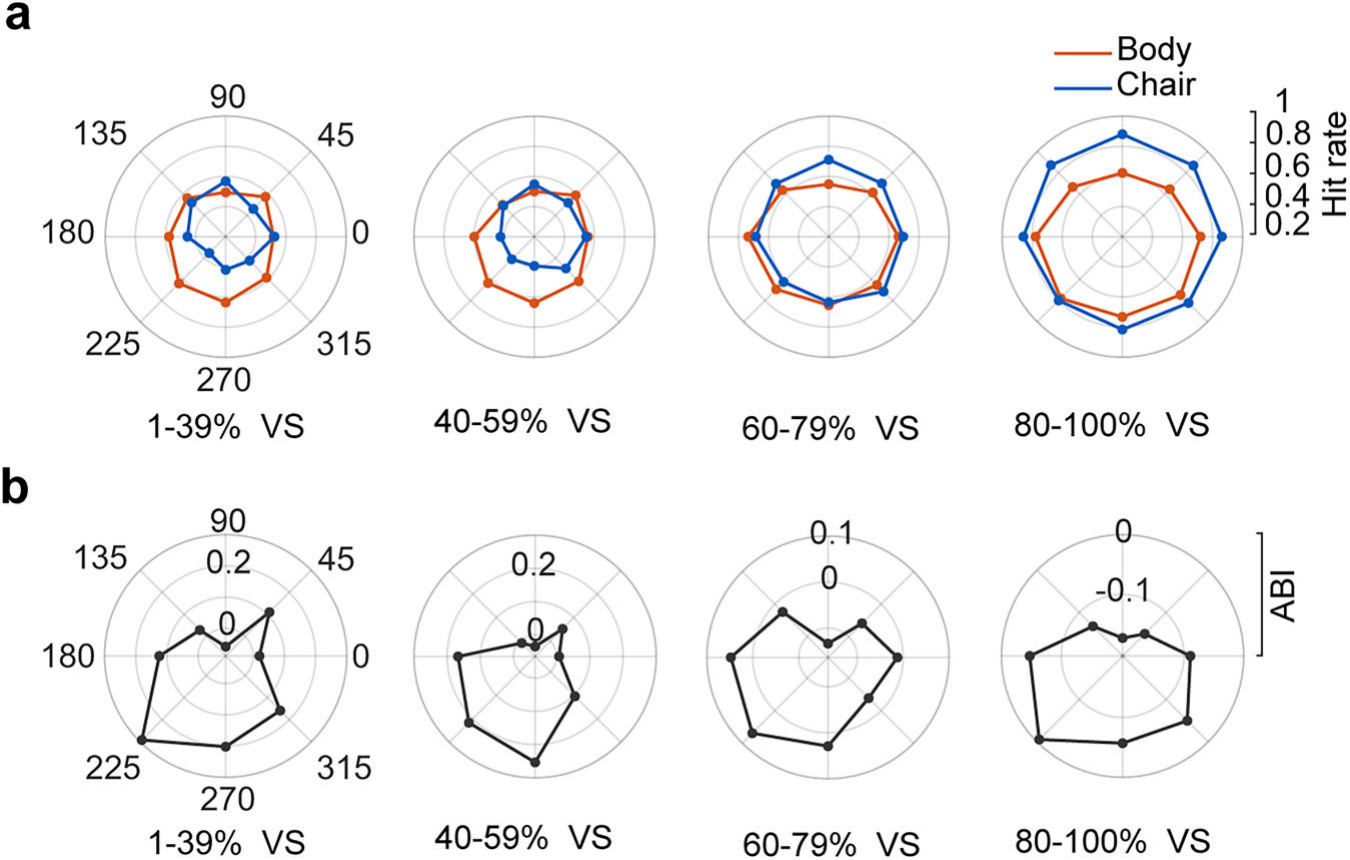
Mean hit rate as a function of stimulus location in the visual field. There was a bias in category detection across different regions of the visual field, as indicated by the computed ABI (animacy bias index). ***a***, Subplots represent the average hit rate of all subjects. Chair is plotted in blue and body in red. The hit rate values are depicted in the right plot. ***b***, The average ABI is plotted for different levels of the visual signals. Each radar plot corresponds to one visual signal.

To further examine the observed bias in category detection in different parts of the visual field, we created radar plots of the average hit rates across the visual field (Fig. 4*a*).

To compute the spatial inhomogeneity in the detection of body compared with the chair, we defined the animacy bias index (ABI; see Materials and Methods). ABI quantifies the normalized bias in body detection. A high ABI value represented a higher tendency to report the body than chair category. Consistent with previously reported spatial bias (Fig. 3), we observed high ABI values in the lower corner and lower-left visual quadrant (Fig. 4*b*, ${\;\mathrm{maximum}\;\mathrm{ABI}}_{\mathrm{visual}\;\mathrm{signal}}^{\mathrm{location}}$: ${\mathrm{ABI}}_{1-39\text{\%}}^{225^{\circ }}\;$ = 0.29 ± 0.06; ${\mathrm{ABI}}_{40-59\text{\%}}^{270^{\circ }}\;$ = 0.24 ± 0.08; $\;{\mathrm{ABI}}_{60-79\text{\%}}^{225^{\circ }}\;$ = 0.07 ± 0.05; ${\mathrm{ABI}}_{80-100\text{\%}}^{225^{\circ }}\;$ = −0.02 ± 0.02).

**Figure 5.**
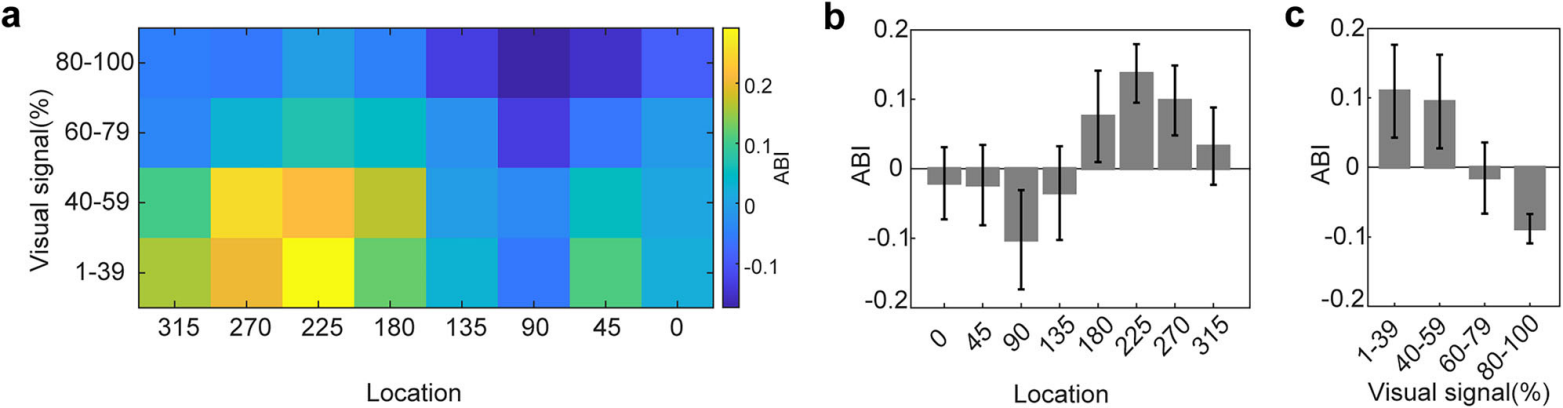
Relationship between visual signal and location of stimulus presentation. There were high positive ABI values in the lower-left visual quadrant at low levels of visual signals. ***a***, Color plot of average animacy bias for all subjects illustrates the influence of location and visual signals on category detection bias. The mean and SEM of animacy bias are plotted as a function of location (***b***) and visual signal (***c***).

To examine the interaction between the influence of location and visual signals on category detection bias, we computed the ABI as a function of the change in visual signals and locations for all subjects (Fig. 5*a*). We found high positive ABI values in the lower-left and low level of visual signals (1–39% visual signal, location 225°: ABI = 0.29 ± 0.06; Fig. 5*a*). In addition, negative ABI values (chair category biased) were observed in the upper-right and high level of visual signals (80–100% visual signal, location 90°: ABI = −0.15 ± 0.26; Fig. 5*a*). A significant systematic change of ABI from a positive value (body bias) to a negative value (chair bias) was observed across different visual signals (Fig. 5*b*, *F*_(3,384)_ = 7.77, *p* < 10^−5^, two-way ANOVA). In addition, a significant change of ABI in the lower field locations compared with upper field locations confirmed the impact of stimulus locations on object recognition (Fig. 5*c*, *F*_(7,384)_ = 3.65, *p* = 0.001, two-way ANOVA). Finally, we observed no interaction between location and the level of visual signal (Fig. 5*a*, *F*_(21,384)_ = 0.33, *p* = 0.994, two-way ANOVA). Table 3 presented the average hit rate, ABI, and *d*′ values in greater detail for each location within the visual field and each visual signal. This detailed breakdown highlighted the directional biases for body and chair detection.

**Table 3.** Average hit rate, animacy bias index (ABI), and *d*′ value in each visual signal and location of visual field


Visual signal	Location	Mean hit rate ± SEM (body)	Mean hit rate ± SEM (chair)	ABI ± SEM	*d*′ value
1–39%	0°	0.53 ± 0.04	0.49 ± 0.05	0.05 ± 0.09	−0.03
	45°	0.55 ± 0.04	0.44 ± 0.04	0.10 ± 0.08	0.58
	90°	0.54 ± 0.04	0.50 ± 0.04	0.04 ± 0.07	−0.33
	135°	0.56 ± 0.04	0.49 ± 0.04	0.05 ± 0.07	0.20
	180°	0.59 ± 0.04	0.40 ± 0.04	0.19 ± 0.08	0.55
	225°	0.64 ± 0.03	0.36 ± 0.03	0.28 ± 0.06	1.84
	270°	0.61 ± 0.04	0.41 ± 0.03	0.18 ± 0.07	1.44
	315°	0.58 ± 0.04	0.46 ± 0.05	0.11 ± 0.09	0.76
40–59%	0°	0.55 ± 0.04	0.52 ± 0.04	0.03 ± 0.08	0.06
	45°	0.58 ± 0.04	0.50 ± 0.05	0.08 ± 0.08	0.32
	90°	0.54 ± 0.04	0.53 ± 0.04	0.02 ± 0.08	−0.23
	135°	0.53 ± 0.04	0.48 ± 0.04	0.05 ± 0.08	0.04
	180°	0.59 ± 0.04	0.43 ± 0.04	0.16 ± 0.07	0.88
	225°	0.66 ± 0.04	0.40 ± 0.03	0.24 ± 0.06	1.47
	270°	0.64 ± 0.05	0.40 ± 0.07	0.24 ± 0.08	1.45
	315°	0.60 ± 0.05	0.50 ± 0.05	0.09 ± 0.08	0.70
60–79%	0°	0.65 ± 0.03	0.71 ± 0.04	−0.03 ± 0.05	−0.21
	45°	0.61 ± 0.04	0.71 ± 0.04	−0.07 ± 0.05	−0.52
	90°	0.58 ± 0.04	0.71 ± 0.05	−0.10 ± 0.08	−0.82
	135°	0.65 ± 0.04	0.71 ± 0.06	−0.03 ± 0.08	−0.28
	180°	0.74 ± 0.03	0.70 ± 0.04	0.04 ± 0.06	0.29
	225°	0.70 ± 0.03	0.61 ± 0.04	0.07 ± 0.05	0.44
	270°	0.68 ± 0.03	0.65 ± 0.05	0.03 ± 0.06	0.12
	315°	0.65 ± 0.04	0.71 ± 0.04	0.04 ± 0.06	−0.36
80–100%	0°	0.72 ± 0.02	0.86 ± 0.03	−0.09 ± 0.02	−1.23
	45°	0.67 ± 0.02	0.87 ± 0.02	−0.12 ± 0.02	−2.55
	90°	0.65 ± 0.03	0.87 ± 0.02	−0.15 ± 0.03	−2.15
	135°	0.68 ± 0.04	0.88 ± 0.02	−0.13 ± 0.03	−1.56
	180°	0.80 ± 0.02	0.86 ± 0.02	−0.03 ± 0.02	−0.69
	225°	0.77 ± 0.02	0.81 ± 0.02	−0.02 ± 0.02	−0.16
	270°	0.73 ± 0.02	0.84 ± 0.02	−0.07 ± 0.02	−0.77
	315°	0.72 ± 0.03	0.84 ± 0.02	−0.08 ± 0.03	−0.61

In addition to comparing the visual field and visual signals, we will also use an ANOVA test to analyze the relationship between the visual field and object categories with the highest visual signals in eight locations. The analysis revealed a significant main effect of visual field for animal body (*F*_animal body(7,120)_ = 4.15, *p* < 10^−3^; *F*_chair(7,120)_ = 0.98, *p* = 0.4; one-way ANOVA) and object category (*F*_category(1, 254)_ = 76.59, *p* < 10^−10^, one-way ANOVA). There is also a significant interaction between visual field and object category (*F*_interaction(7, 240)_ = 3.99, *p* < 10^−3^, two-way ANOVA).

**Figure 6.**
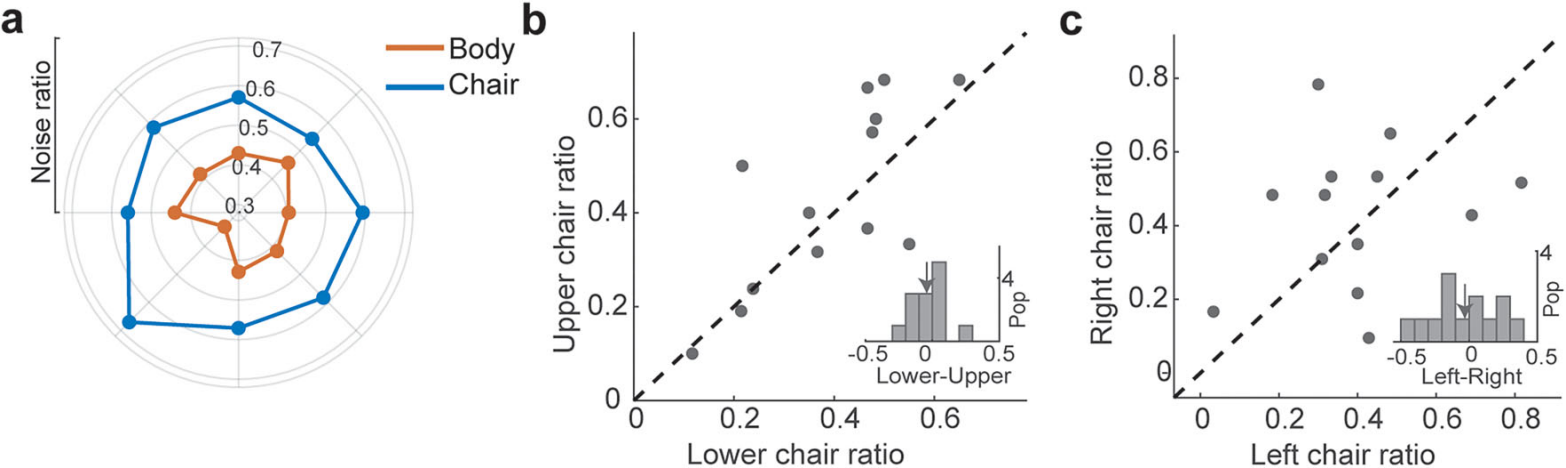
Individual subjects’ hit rate for full noise stimuli. There was no significant difference in full noise bias across locations. The performance of chair and body (expressed as the ratio of reported body/chair trials to all trials) in response to full noise stimuli is plotted for each location (***a***). The scatter plots of chair ratio for (***b***) lower versus upper and (***c***) left versus right visual field stimuli are plotted for all subjects for the full noise stimuli (*n* = 13; the full noise stimuli were not presented to the three subjects). The histograms on the scatter plots illustrate the distribution of the chair ratio differences.

To control the potential contribution of higher cognitive functions such as spatial attention and task demand, on the response bias, we plotted the subjects’ chair hit rate for the full noise stimuli. We have plotted the hit rate of both the chair and body conditions (expressed as the ratio of reported body/chair trials to the total number of trials) in response to full noise stimuli for each respective location (Fig. 6*a*). The ANOVA shows no significant difference in full noise bias across locations (*p* = 0.91). Also, we found no significant difference in the upper and the lower as well as right and left visual hemifield (Fig. 6*b*,*c*, mean chair hit rate ± SEM in the full noise stimuli: lower, 0.38 ± 0.03; upper, 0.41 ± 0.04, *p*_(lower, upper)_ = 0.44; left, 0.38 ± 0.03; right, 0.42 ± 0.05, *p*_(left, right)_ = 0.54).

**Figure 7.**
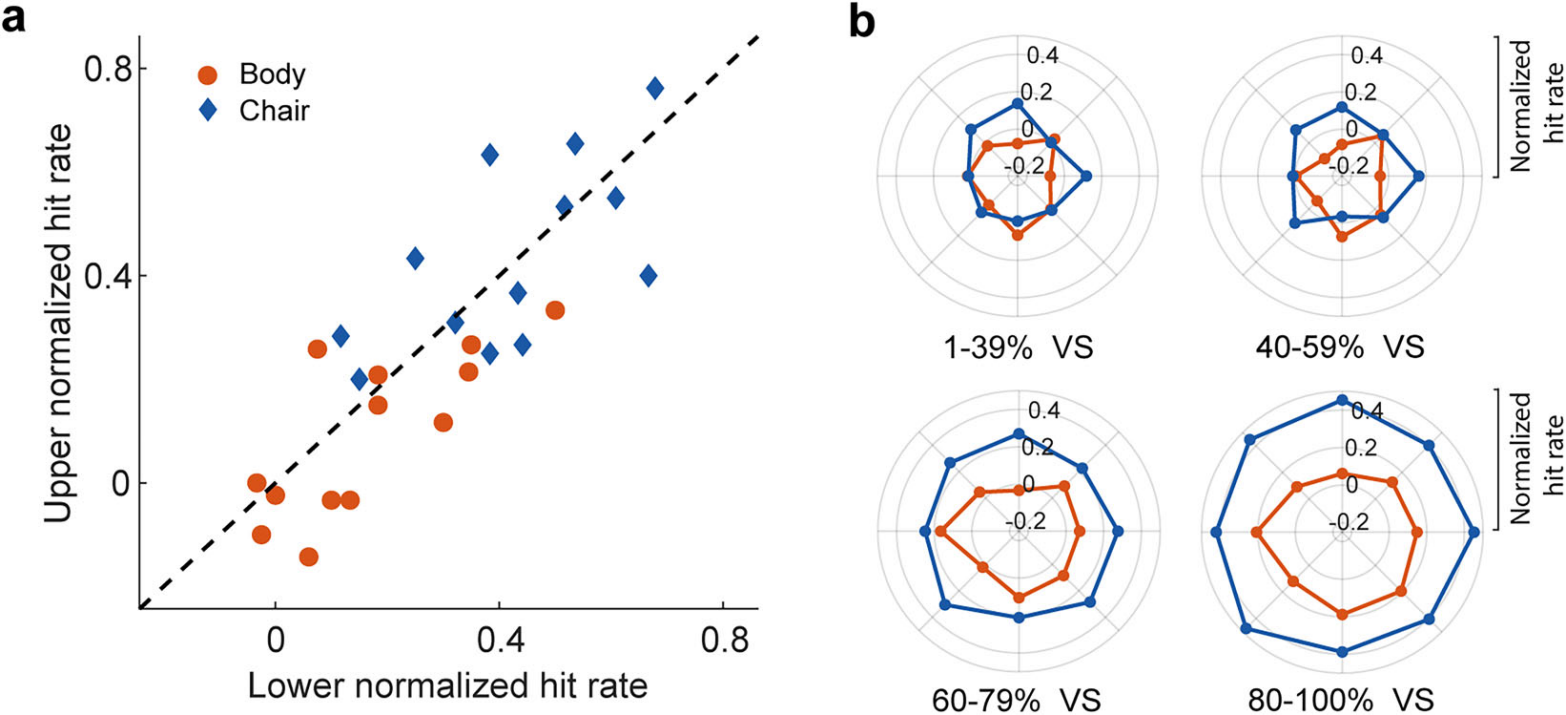
Body and chair category recognition across signal levels and locations after correction for location-contingent decision bias. Heterogeneity persisted in body category recognition, while normalized detection showed no bias toward chairs. The left panel (***a***) depicts the results of category recognition after correcting for the full noise detection bias for 80–100% visual signal. The right panel (***b***) shows the bias-corrected category recognition for both body and chair categories at different signal level covering.

Despite the absence of significant differences between upper and lower locations for body and chair detection in full noise conditions, a numerical trend emerged. To address this, we assumed that response bias remained consistent in full noise conditions. We normalized category detection by subtracting full noise detection levels to mitigate the influence of response bias on reported outcomes. Figure 7*a* illustrates that the heterogeneity endured in body category recognition, while normalized detection exhibited no chair bias. These findings imply that while heterogeneous response biases exist in both body and chair categorization, only body category recognition is influenced by the stimulus’ visual field location (Fig. 7*a*, scatter plot; chair, *p* = 0.78; body, *p* = 0.04). Figure 7*b* displays the normalized body and chair detection levels across all signal intensities and locations.

**Figure 8.**
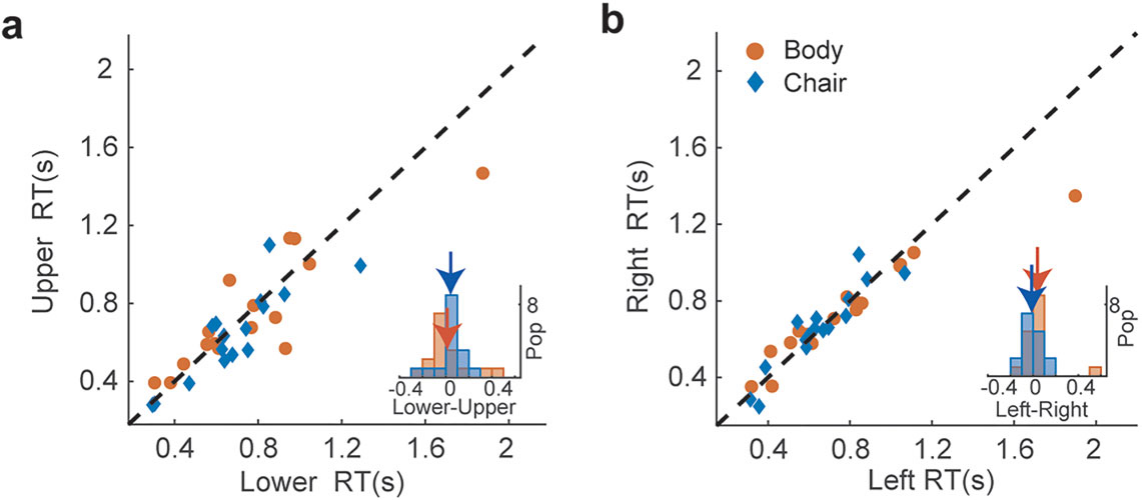
Individual subjects’ reaction times for detecting body and chair categories in the high level of visual signal (80–100%). Category recognition for both body and chair categories resulted in similar reaction times across various locations in the visual field. The scatter plot of reaction times for category detection in the (***a***) lower versus upper and (***b***) right versus left visual field stimuli. The bottom right histograms show the cumulative distribution of the difference between the lower/upper and right/left visual field reaction times.

Furthermore, we compared subjects’ reaction times across different parts of the visual field for the less noisy stimuli (80–100% visual signal). Although the chair category elicited quicker responses than the bodies category (*p* *=* 0.002), we observed similar reaction times for category recognition across different locations of the visual field for both body and chair categories [Fig. 8*a*,*b*; mean (RT) ± SEM (RT): ${\mathrm{RT}}_{\mathrm{animal}}^{\mathrm{lower}}$ = 0.76 ± 0.09 s, ${\mathrm{RT}}_{\mathrm{animal}}^{\mathrm{upper}}$ = 0.75 ± 0.06, *p* = 0.79/ ${\mathrm{RT}}_{\mathrm{chair}}^{\mathrm{lower}}$ = 0.68 ± 0.06, ${\mathrm{RT}}_{\mathrm{chair}}^{\mathrm{upper}}$ = 0.64 ± 0.06, *p* = 0.09/ ${\mathrm{RT}}_{\mathrm{animal}}^{\mathrm{left}}$ = 0.77 ± 0.09, ${\mathrm{RT}}_{\mathrm{animal}}^{\mathrm{right}}$ = 0.73 ± 0.07, *p* = 0.47/ ${\mathrm{RT}}_{\mathrm{chair}}^{\mathrm{left}}$ = 0.67 ± 0.06, ${\mathrm{RT}}_{\mathrm{chair}}^{\mathrm{right}}$ = 0.65 ± 0.05, *p* = 0.80].

## Discussion

Here, we showed that the category recognition performance for body and chair stimuli varied across the visual field. The hit rate was lower when stimuli were presented outside the fovea. The largest decline was observed when body images were presented in the upper visual field. This bias in body recognition was more pronounced in the lower-left visual field and for noisier images.

Most of the primates’ visual neural resources are devoted to the processing of stimuli that appear at the center of the visual field. Therefore, objects located at the center of the visual field are recognized far better than those in the peripheral vision (Rosenholtz, 2016; Stewart et al., 2020). However, behavioral and electrophysiological studies that have addressed the mechanisms of peripheral object vision point to the significance of peripheral vision object recognition (Op De Beeck and Vogels, 2000; Harrison and Bex, 2015; Chen et al., 2019; Kar et al., 2019; Ramezani et al., 2019). For example, peripheral visual information improves stimulus detection at the center of the gaze (Henderson and Anes, 1994) and plays a significant role in spatial attention, visual search, and control of eye movements (Rosenholtz et al., 2012). Bias in category representation across the visual field has been reported in previous studies (Rijsdijk et al., 1980; Cannon, 1985; Carrasco et al., 2001; Legge et al., 2001). This bias seems to depend on the task (Thomas and Nicholls, 2018; Himmelberg et al., 2020) and the tested stimulus category (Hübner et al., 1985; Mäkelä et al., 2001; Martelli et al., 2005; Hershler et al., 2010). Object recognition is also contingent on the location of objects relative to the horizontal and vertical meridian boundaries (Quek and Finkbeiner, 2014, 2016; de Haas et al., 2016; Ghafari et al., 2022). This location-dependent object recognition seems to emerge during the early stages of development and depends on the statistics of sensory experience for each specific stimulus across the visual field (Tsurumi et al., 2023). It has previously been shown that body detection remains above chance level even at 70° eccentricities (Thorpe et al., 2001). Also, recognition performance is better for faces and animal bodies compared with cars presented at eccentricities <10° over the horizontal meridian (Boucart et al., 2016). In line with these studies, our results also focus on the perception of objects outside the fovea and suggest the perceptual heterogeneity in distinguishing between chairs and living things at different visual field locations.

The nature of this variability for object recognition across locations is well studied for faces (Mäkelä et al., 2001; Afraz et al., 2010), but to the best of our knowledge, potential differences in nonface recognition across the visual field quadrants have not been the focus of research studies. Our study reveals the differences in the body and chair recognition at different extrafoveal locations and characterizes a bias toward body compared with chair detection that varies across the visual field.

The asymmetrical distribution of attention in different areas of the visual field can be one of the factors causing the observed results. However, differences in attentional deployment usually affect response time. In our experiments, similar reaction times for chair and body categories (Fig. 8) suggest that differences in brain states, such as the level of attention and alertness, could not have induced our observed results. Furthermore, similar subjects’ performance for the full noise stimuli at different extrafoveal locations (Fig. 6) suggests that the observed bias in category recognition at different visual field locations was category-specific.

The low accuracy of object recognition in the low signal level conditions, as clearly observed in Figure 3, may be attributed to the presence of noisy peripheral targets. However, our study revealed a systematic response bias that changed across noise levels for both body and chair stimuli. Additionally, as depicted in Figure 5, our results revealed a double dissociation where there was a bias toward animal bodies in the lower visual field in stimuli with low visual signals and chairs in the upper visual field in stimuli with high visual signals. These findings underscore the reliability of observer response bias in noisy conditions.

To account for a potential location-contingent decision bias on the category recognition, we corrected the hit rate by subtraction of the full noise hit rates from the category hit rates (Fig. 7). Location-dependent category recognition was observed only for body category. These results suggest heterogeneous body recognition bias across the visual field potentially due to more frequent exposure of the lower visual field to body stimuli.

The discrepancy between body and chair reports is evident in the full noise condition (Fig. 6), aligning with our observations from both low and high noise stimuli. In cases of noisier stimuli, there is a tendency for body-specific detection to be triggered in the lower visual field. Furthermore, the physical constraints related to body location given the more frequent exposure of body images in the lower peripheral field when focusing on faces. In addition, the dominance of the right hemisphere in visual integration (Joseph, 1988) and body detection could contribute to the observed bias. To explore these possibilities more comprehensively, further studies incorporating a larger sample size and a broader array of stimuli would be invaluable.

To enhance the generalizability of observed heterogeneity, it is advantageous to utilize a broader range of visual stimuli, encompassing both an increased number of images and diverse categories. Additionally, varying eccentricity can contribute to this generalization across the visual field. Achieving this objective necessitates the execution of further experiments. 

Research demonstrates that the variation in object categorization across the visual field is attributable to the inherently inhomogeneous nature of high-level visual representations. Although current convolutional neural networks impose homogeneous category representations across the visual field, existing brain-based evidence emphasizes that category-selective regions in the high-level visual cortex do not evenly sample the visual field (Kay et al., 2015; Silson et al., 2016; Le et al., 2017; Poltoratski et al., 2021). Our results align with this research, reinforcing the idea that high-level visual representations are inhomogeneous across visual space. This correspondence between behavioral observations and neural processing supports our understanding of visual perception and the organization of the visual cortex, highlighting the complexity of object recognition in various visual contexts.

The observed heterogeneity of category recognition can be explained by the fact that neural sampling is sparse. This is because even at high levels of the visual system, stimuli are analyzed by cells with relatively limited receptive field sizes that do not cover the entire visual field. Additionally, a stimulus only activates a few cells or groups of cells. This sparse sampling can manifest itself in local bias (Legge et al., 2001).

Research indicates that animals, as visual stimuli, are processed differently from human bodies, particularly by subcortical structures such as the amygdala, which plays a critical role in visual perception (Almeida et al., 2015; Šimić et al., 2021). This differential processing may influence how we perceive and respond to animal bodies compared with human bodies (Peelen and Downing, 2007). Although animal bodies statistically appear in the lower visual field similarly to human bodies, the distinct neural pathways and cortical representations involved suggest that our perception of these two categories of bodies may diverge significantly.

Here, we extend the current knowledge about category recognition across the different visual fields and show the perceptual heterogeneity of high-level visual processing in the peripheral visual field. Our observations suggest that the special cortical mechanism also exists for peripheral vision in higher cortical areas. Our results shed light on the heterogeneity of the processing of different categories across the visual field. This nonuniformity in the processing of objects should be considered in future studies and models of object recognition.

## Synthesis

Reviewing Editor: Ifat Levy, Yale School of Medicine Decisions are customarily a result of the Reviewing Editor and the peer reviewers coming together and discussing their recommendations until a consensus is reached. When revisions are invited, a fact-based synthesis statement explaining their decision and outlining what is needed to prepare a revision will be listed below. The following reviewer(s) agreed to reveal their identity: NONE.

This study examined visual object recognition at different locations of the visual field, using a two-alternative forced-choice category-recognition task with images of chairs and animal bodies at different levels of visual ambiguity. The authors report differences in performance between categories as a function of visual field location, with animal body stimuli showing a lower visual field bias that persists even when correcting for general response bias in noise stimuli.

The paper addresses an interesting topic, and the results appear clean and relatively straightforward. Reviewers identified, however, several issues that require clarification, as detailed below.

- Many details of the statistical analysis are missing or not clear:

o How exactly was hit rate calculated (page 6-7)

o Please provide information as to the software that was used to perform the statistical analyses and indicate the specific functions used.

o There are multiple places where statistical information is not fully provided. Please use the recommended reporting conventions: when reporting the two-sided Wilcoxon's signed-rank test include the test statistic with the degrees of freedom. Also, when reporting ANOVA include the F value with degrees of freedom, the factors and levels of each factor and information regarding repeated/non-repeated measures should all be reported.

o It is unclear in the methods whether "performance" was calculated as the hit rate. It is stated that hit rate was calculated across all positions, then later the ABI is defined, but performance in reference to ABI is not defined. Could you clarify if it's hit rate, D-prime, etc.

o The comparisons in the left/right visual field consist of multiple tests (presumably t-tests?). It seems more appropriate to run an ANOVA with factors of visual field and object category. It might be best to perform the statistical analysis with an ANOVA before testing specific locations with t-tests.

o The stats in the results section of Figure 3 are not defined, are these paired t-tests? If so please report the t-values and the degrees of freedom, and specify the statistical test being run.

o Can the authors report d-prime values at the different visual field locations as a supplementary figure? This is a pretty standard psychophysical metric, which many readers would appreciate.

o For some delta results, both average and SEM are reported, but for others only the average is reported. Please add SEM to all reports.

- The authors should clarify early in the manuscript that the "bodies" are animal bodies. It is possible that animals, as a visual stimulus, are treated differently than humans, particularly by subcortical structures like the amygdala which might orient our attention. Although statistically animal bodies likely appear in the lower visual field, similar to human bodies, the authors may want to discuss this difference, and any potential differences in cortical representations of human and animal bodies.

- Please define "upper visual field" and "lower visual field" before first using the terms. Is it everything above or below the horizontal meridian (I.e., the 3 upper locations in Figure 2C are considered upper visual field)? or is it just the one highest or lowest location? Labeling this directly in Figure 2, in addition to specifying within the text will be helpful.

- It would be helpful if the authors added to the figure legends explanations as to how the results in each figure support the findings reported in the text to assist in readability.

- Figure 2b: for the lower visual field comparison there are ** above the bars. However, from the text it seems that the p-value is 0.09 (page 8)? Please explain which statistical analysis refers to the plot.

- In Page 8 of the results the authors say that "comparing average performance in the lower and upper extra foveal location showed chair performance was better than body detection". It should be clarified that ABI was used to calculate this difference. The methods describe how ABI is derived, but the results do not mention what metric was used to measure this difference. Furthermore, the difference is not significant so the statement should be toned down.

- Could you provide some more explanation of what exactly is presented in Figure 2c, and how the results presented there are consistent with Table S1?

- Direct labeling of the rows of Figure 4 will be very helpful.

- The authors state that "The lower/upper field bias of body/chair detection was observed at all levels of the visual signal (Table S2)". This seems inconsistent with Figure 2a, where the blue chair performance appears higher at both the upper and lower vertical meridian positions at the highest level of visual signal. Please explain.

- Table S1 presents the across-participants average of performance ranges - please add the SEM across participants.

- Introduction: Additional studies investigating category related perception across the visual field that may also be cited are:

o Kreichman, Bonneh, and Gilaie-Dotan (2020) Investigating face and house discrimination at foveal to parafoveal locations reveals category-specific characteristics. Scientific Reports 10: 8306

o Morsi, Goffaux, and Greenwood (2024) The resolution of face perception varies systematically across the visual field. Plos One 19: e0303400

- In the discussion the authors state that "we must revise our classical view of hierarchical feedforward processing in the brain"; this does not seem justified. The authors base their argument on neural networks, rather than on existing brain-based evidence. Receptive field mapping, for example, has shown for decades that category representations are not homogeneous across the visual field. The following papers are examples of research showing that faces, places, and words are category-selective regions in high-level visual cortex that do not evenly sample the visual field. Thus, the notion that we need to rethink how visual cortex is organized is a straw-man argument that should probably be removed. Instead, the authors can write that their results are in line with research showing that high-level visual representations are inhomogeneous across the visual space, so the correspondence between behavior and brain function is in line with our current theories of vision (which is great!).

o "The field of view available to the ventral occipito-temporal reading circuitry" by Le, Witthoft, Ben-Shachar, and Wandell. Journal of Vision, 2017.

o "Attention reduced spatial uncertainty in human ventral temporal cortex" by Kay, Weiner, and Grill-Spector. Current Biology, 2015.

o "Evaluating the correspondence between face-, scene-, and object-selectivity and retinotopic organization within lateral occipitotemporal cortex" buy Wilson, Groen, Kravtiz, and Baker. Journal of Vision, 2016.

o "Holistic face recognition is an emergent phenomenon of spatial processing in face-selective regions" Poltoratski, Kay, Finzi, Grill-Spector. Nature Communications, 2021.

Minor comments:

- Page 5. Participants: 'The test was conducted without any intervention in the process.'

What do the authors mean by 'intervention'?

- Page 6. Data analysis: '...across all trails...' instead of trials.

- Page 9. The two sentences describing Figure S1 seem to repeat the same conclusion:

o 'There was similar upper/lower visual field bias for chair/body category in other levels of the visual signal.'

o 'The lower/upper field bias of body/chair detection was observed across all levels of the visual signal (Table S2).'

- Page 12. 'This location dependent object recognition seems to emerge during early stages of development and depends on the statistics of sensory experience for each specific stimulus across the visual field (Tsurumi et al., 2021)'.

The reference provided (Tsurumi et al., 2021) is a Vision Sciences Society Annual Meeting Abstract, however, a paper following this abstract has already been published: Tsurumi et al.,2023 (Development of upper visual field bias for faces in infants. Developmental Science).

- Missing space before parenthesis:

o Page 14. '...homogeneity of representation across the visual field(Stewart et al., 2020)'.

o Page 15. '...manifest itself in local bias(Legge et al., 2001b)'.

- References: some references appear twice, for example: Barbot A, Xue S, Carrasco M (2021a); Himmelberg MM, Winawer J, Carrasco M (2020a); Cannon MW (1985a); Legge GE, Mansfield JS, Chung STL (2001a); Quek GL, Finkbeiner M (2016a); Thomas NA, Nicholls MER (2018a).

## Author Response

Dear Dr. Ifat Levy, We have revised our manuscript, titled "Heterogeneity in Category Recognition across the Visual Field," # eN-NWR-0331-24, in response to the reviewers' comments. We appreciate their valuable feedback, as addressing these points has greatly enhanced the clarity and rigor of the manuscript. Below, we provide point-by-point responses to each of the reviewers' concerns.

Additionally, we would like to note that, given the restriction on supplementary materials, we have omitted Figure S1 and included the relevant data in Table 2 to maintain clarity within the main text. We have also renamed Table S1 to "Table 1" and Table S2 to "Table 3"." Synthesis of Reviews:

Computational Neuroscience Model Code Accessibility Comments for Author (Required):

Please include a statement in the Materials and Methods section, under the heading "Code Accessibility", indicating whether and how the code can be accessed, including any accession numbers or restrictions, as well as the type of computer and operating system used to run the code.

Synthesis Statement for Author (Required):

This study examined visual object recognition at different locations of the visual field, using a two-alternative forced-choice category-recognition task with images of chairs and animal bodies at different levels of visual ambiguity. The authors report differences in performance between categories as a function of visual field location, with animal body stimuli showing a lower visual field bias that persists even when correcting for general response bias in noise stimuli.

The paper addresses an interesting topic, and the results appear clean and relatively straightforward. Reviewers identified, however, several issues that require clarification, as detailed below.

Many details of the statistical analysis are missing or not clear:

How exactly was hit rate calculated (page 6-7) We calculated the hit rate using the following formula:

Hit Rate = (Number of Correct Responses / Total Number of Stimuli) × 100.

In our study, a "hit" was defined as a correct identification or response to a stimulus. The total number of stimuli includes both animal body and chair stimuli presented during the experiment. By dividing the number of correct responses (hits) by the total stimuli and multiplying by 100, we obtained the percentage of correct responses, which we refer to as the hit rate. We modified explanation of hit rate in method, data analysis section.

The added and revised text in the manuscript is as follows: "We calculated the hit rate by assessing the subject's ability to correctly detect target stimuli (animal body or chair categories) across all trials for each level of visual signal at different locations. For each visual signal level, we measured the ratio of correct responses (i.e., trials where the subject accurately identified the target category-either body or chair) to the total number of trials presented at that signal level. Specifically, for the condition with the highest noise (i.e., the full noise level), the hit rate was calculated as the ratio of trials where the subject reported detecting the body or chair category to the total number of trials presented under this condition." We also changed all performance and perf to hit rate in manuscript and highlighted them.

Please provide information as to the software that was used to perform the statistical analyses and indicate the specific functions used.

We used MATLAB software along with its Statistical Toolbox to perform the statistical analyses. This information has been included in the manuscript, where we detail the software and the specific functions employed. We added software and details of code and functions we used in code accessibility and the Method section. The added text in the manuscript is as follows: "All statistical analyses were conducted using MATLAB software with the Statistical Toolbox." There are multiple places where statistical information is not fully provided. Please use the recommended reporting conventions: when reporting the two-sided Wilcoxon's signed-rank test include the test statistic with the degrees of freedom. Also, when reporting ANOVA include the F value with degrees of freedom, the factors and levels of each factor and information regarding repeated/non-repeated measures should all be reported.

For the two-sided Wilcoxon signed-rank test, we used sixteen samples, which required the use of the exact method. As a result, the degrees of freedom are not reported, as they are only applicable to the approximate method. For the ANOVA test, we have included both the F-value and degrees of freedom in the manuscript. The added statistics and degrees of freedom are as follows: "(Figure 5b, F(3,384) = 7.77, p<10-5, two-way ANOVA). (Figure 5c, F(7,384) = 3.65, p= 0.001, two-way ANOVA). (Figure 5a, F(21,384) = 0.33, p = 0.994, two-way ANOVA)." It is unclear in the methods whether "performance" was calculated as the hit rate. It is stated that hit rate was calculated across all positions, then later the ABI is defined, but performance in reference to ABI is not defined. Could you clarify if it's hit rate, D-prime, etc.

To calculate the ABI index, we used the hit rate ratio as a measure of the subject's performance. For clarity, we have updated all instances of 'performance' to 'hit rate' throughout the manuscript.

The comparisons in the left/right visual field consist of multiple tests (presumably t-tests?). It seems more appropriate to run an ANOVA with factors of visual field and object category. It might be best to perform the statistical analysis with an ANOVA before testing specific locations with t-tests.

To compare left/right and upper/lower hemifields, we averaged the locations within each hemifield. As a result, we did not have multiple conditions, so we used the Wilcoxon signed-rank test. In Figure 5, we compared multiple conditions involving location and visual signal using a two-way ANOVA test. Based on your suggestion, in addition to comparing visual field and visual signal, we will also compare visual field and object category using an ANOVA test. The added statistics analysis of ANOVA test are as follows: "In addition to comparing visual field and visual signal, we will also compare visual field and object category using an ANOVA test. The analysis revealed a significant main effect of visual field for animal body (Fanimal body(7,120) = 4.15, p < 10-3, Fchair(7,120) = 0.98, p =0.4, one-way ANOVA) and object category (Fcategory(1, 254) = 76.59, p < 10-10one-way ANOVA). There is also significant interaction between visual field and object category (Finteraction(7, 240) = 3.99, p <10-3, two-way ANOVA." The stats in the results section of Figure 3 are not defined, are these paired t-tests? If so please report the t-values and the degrees of freedom, and specify the statistical test being run.

As stated in the Statistical Analysis section, a two-sided Wilcoxon signed-rank test was used to calculate statistical significance in all analyses unless otherwise stated.' For Figure 3a, we also used the two-sided Wilcoxon signed-rank test, which has been added to the text. Due to the sample size (sixteen subjects), we applied the exact method, so the degrees of freedom cannot be reported.

Can the authors report d-prime values at the different visual field locations as a supplementary figure? This is a pretty standard psychophysical metric, which many readers would appreciate.

Thank you for your suggestions. We have added the d-prime values to Table 3 and included the method for calculating d-prime in the Analysis section. The updated table is as follows:

For some delta results, both average and SEM are reported, but for others only the average is reported. Please add SEM to all reports.

I have added the standard error of the mean (SEM) to all reported values. The revised manuscript now includes the SEM values.

The authors should clarify early in the manuscript that the "bodies" are animal bodies. It is possible that animals, as a visual stimulus, are treated differently than humans, particularly by subcortical structures like the amygdala which might orient our attention. Although statistically animal bodies likely appear in the lower visual field, similar to human bodies, the authors may want to discuss this difference, and any potential differences in cortical representations of human and animal bodies.

To remove any ambiguity, we specified that the 'bodies' refer to animal bodies in the abstract and throughout various sections of the manuscript. To further clarify the perceptual differences between human and animal bodies, we have added an explanation in the discussion addressing these differences and their implications for cortical representations. The added text in the Discussion is as follows: "Research indicates that animals, as visual stimuli, are processed differently from human bodies, particularly by subcortical structures such as the amygdala, which plays a critical role in visual perception (Almeida et al., 2015; Šimić et al., 2021). This differential processing may influence how we perceive and respond to animal bodies compared to human bodies (Peelen et al., n.d.). Although animal bodies statistically appear in the lower visual field similarly to human bodies, the distinct neural pathways and cortical representations involved suggest that our perception of these two categories of bodies may diverge significantly." Please define "upper visual field" and "lower visual field" before first using the terms. Is it everything above or below the horizontal meridian (I.e., the 3 upper locations in Figure 2C are considered upper visual field)? or is it just the one highest or lowest location? Labeling this directly in Figure 2, in addition to specifying within the text will be helpful.

To clarify the definitions of 'upper visual field' and 'lower visual field,' we refer to the three locations above and below the horizontal meridian line, respectively. Thus, the three upper locations in Figure 2C are considered part of the upper visual field, while the three lower locations are classified as the lower visual field. We added labels directly to Figure 2 to enhance clarity and will specify these definitions in the text before first using the terms. Additionally, we have included an explanation of the exact locations for the upper/lower and right/left visual fields in the Methods section as follows: "The upper/lower visual fields correspond to three locations above/below the horizontal meridian: the upper visual field includes visual angles of 45{degree sign}, 90{degree sign}, and 135{degree sign}, while the lower visual field includes 225{degree sign}, 270{degree sign}, and 315{degree sign}. The right/left visual fields are defined as the locations to the right/left of the vertical meridian: the right visual field includes angles of 315{degree sign}, 0{degree sign}, and 45{degree sign}, while the left visual field includes 135{degree sign}, 180{degree sign}, and 225{degree sign}." It would be helpful if the authors added to the figure legends explanations as to how the results in each figure support the findings reported in the text to assist in readability.

Thank you for your suggestion regarding the figure legends. We have revised all legends and added a few sentences to explain the main messages and results of each figure. The updated figure legends are as follows: "Figure 2. Differences in subjects' hit rate for body and chair categories. Subjects' performance in detecting body and chair categories varies between foveal and extrafoveal locations.

Figure 3. Subjects' hit rate to detect less noisy stimuli (80-100%) of body and chair. There was variability in responses within the body and chair categories across the extrafoveal locations of the visual field a. Individual subjects' hit rate in the lower and upper visual fields.

Figure 4. Mean hit rate as a function of stimulus location in the visual field. There was a bias in category detection across different regions of the visual field, as indicated by the computed ABI index.

Figure 5. Relationship between visual signal and location of stimulus presentation. There were high positive ABI values in the lower left visual quadrant at low levels of visual signals Figure 6. Individual subjects' hit rate for full noise stimuli. There was no significant difference in full noise bias across locations.

Figure 7. Body and chair category recognition across signal levels and locations after correction for location-contingent decision bias. Heterogeneity persisted in body category recognition, while normalized detection showed no bias toward chairs.

Figure 8. Individual subjects' reaction times for detecting body and chair categories in the high level of visual signal (80-100%). Category recognition for both body and chair categories resulted in similar reaction times across various locations in the visual field Figure 2b: for the lower visual field comparison there are ** above the bars. However, from the text it seems that the p-value is 0.09 (page 8)? Please explain which statistical analysis refers to the plot.

Thanks for your attention. P-value = 0.09 is true and we corrected figure 2.b.

In Page 8 of the results the authors say that "comparing average performance in the lower and upper extra foveal location showed chair performance was better than body detection". It should be clarified that ABI was used to calculate this difference. The methods describe how ABI is derived, but the results do not mention what metric was used to measure this difference. Furthermore, the difference is not significant so the statement should be toned down.

We would like to clarify that the difference mentioned in the results was not computed using ABI. Instead, we used the difference in performance for detecting stimuli. In Figure 2b, we compared the differences between two conditions. The notation ∆_((a,b))^c represents the difference in performance between conditions 'a' and 'b' for situation 'c'. Specifically, we calculated the difference in performance for detecting chairs and animal bodies in the fovea (Figure 2b; 〖 ∆〗_((chair,animal))^fovea = -0.0001, p = 0.9) and then for the upper and lower visual fields (Figure 2b; 〖 ∆〗_((chair,animal))^upper = 0.23, p <10-4; 〖 ∆〗_((chair,animal))^lower = 0.06, p = 0.09). While there is no significant difference in chair and body detection in the fovea, chair detection is significantly better in the extrafoveal presentation, particularly in the upper visual field. We have added an explanation at the beginning of Figure 2 to clarify these differences. The revised text is provided below: "The response curve in Figure 2a shows that subjects' performance in detecting body and chair categories differs between the foveal and extrafoveal locations. To identify these differences, we compared the subjects' hit rate, 〖 ∆〗_((chair,animal))^location , for less noisy stimuli (80-100% visual signal) between the foveal and extrafoveal locations." Could you provide some more explanation of what exactly is presented in Figure 2c, and how the results presented there are consistent with Table S1? Body detection is more sustained than chair detection across different levels of visual signals. To assess the fluctuations in body and chair detection, in Figure 2c, we calculated the maximum and minimum hit rates for body and chair detection across various visual signals for each subject, at each location. The shaded blue and red areas represent the range of minimum to maximum responses across subjects, while the red and blue lines show the average hit rates for body and chair detection at each location. The numerical range from minimum to maximum values is displayed in Table 1. We updated following explanation to Figure 2c to clarify the observed variation.: "Body detection is more sustained than chair detection across different levels of visual signals. To assess the fluctuations in body and chair detection, in Figure 2c, we calculated the maximum and minimum hit rates for body and chair detection across various visual signals for each subject, at each location. The shaded blue and red areas represent the range of minimum to maximum responses across subjects, while the red and blue lines show the average hit rates for body and chair detection at each location. The numerical range from minimum to maximum values is displayed in Table 1." Direct labeling of the rows of Figure 4 will be very helpful.

We have revised Figure 4 and updated the labeling.

The authors state that "The lower/upper field bias of body/chair detection was observed at all levels of the visual signal (Table S2)". This seems inconsistent with Figure 2a, where the blue chair performance appears higher at both the upper and lower vertical meridian positions at the highest level of visual signal. Please explain.

Thank you for your observation regarding the statement about the lower/upper field bias of body/chair detection. As shown in Figure 2b and as you noted, at the highest level of visual signal, chair detection is indeed better than animal detection in both the upper and lower visual fields. In the paper, we highlight the heterogeneity of animal detection by comparing the hit rates of animal detection in the upper and lower fields, as well as the heterogeneity of chair detection across the visual field by comparing the hit rates of chair detection in the upper and lower fields. This heterogeneity refers to the comparison of hit rates within a single category (either animal or chair) in the upper and lower visual fields, rather than comparing chair to animal detection across different locations.

Consequently, we observed a lower visual field bias for body detection compared to the upper field and an upper field bias for chair detection compared to the lower field. This is showed in Table 2 that replaces Figure S2. Also clarify this issue, we have modified the explanation in Table 3 (renamed from Table S2). "We revised explanation of table 3 as follows and also: "The lower field bias for body detection and the upper field bias for chair detection were observed across all levels of visual signal. (Table 2)." "Table 3 presented the average hit rate, ABI, and d' values in greater detail for each location within the visual field and each visual signal. This detailed breakdown highlighted the directional biases for body and chair detection." Table S1 presents the across-participants average of performance ranges - please add the SEM across participants.

We have added the standard error of the mean (SEM) to Table 1 (renamed from Table S1).

Introduction: Additional studies investigating category related perception across the visual field that may also be cited are: o Keichman, Bonneh, and Gilaie-Dotan (2020) Investigating face and house discrimination at foveal to parafoveal locations reveals category-specific characteristics. Scientific Reports 10: 8306 o Morsi, Goffaux, and Greenwood (2024) The resolution of face perception varies systematically across the visual field. Plos One 19: e0303400 We have added these references in the Introduction as follows: ". When comparing face and house discrimination, faces were more affected than houses in parafoveal locations (Kreichman et al., n.d.). Face perception is systematically influenced by the location of face presentation (Morsi et al., 2024)." In the discussion the authors state that "we must revise our classical view of hierarchical feedforward processing in the brain"; this does not seem justified. The authors base their argument on neural networks, rather than on existing brain-based evidence. Receptive field mapping, for example, has shown for decades that category representations are not homogeneous across the visual field. The following papers are examples of research showing that faces, places, and words are category-selective regions in high-level visual cortex that do not evenly sample the visual field. Thus, the notion that we need to rethink how visual cortex is organized is a straw-man argument that should probably be removed. Instead, the authors can write that their results are in line with research showing that high-level visual representations are inhomogeneous across the visual space, so the correspondence between behavior and brain function is in line with our current theories of vision (which is great!). o "The field of view available to the ventral occipito-temporal reading circuitry" by Le, Witthoft, Ben-Shachar, and Wandell. Journal of Vision, 2017. o "Attention reduced spatial uncertainty in human ventral temporal cortex" by Kay, Weiner, and Grill-Spector. Current Biology, 2015. o "Evaluating the correspondence between face-, scene-, and object-selectivity and retinotopic organization within lateral occipitotemporal cortex" buy Wilson, Groen, Kravtiz, and Baker. Journal of Vision, 2016. o "Holistic face recognition is an emergent phenomenon of spatial processing in face-selective regions" Poltoratski, Kay, Finzi, Grill-Spector. Nature Communications, 2021.

We have revised this section to explain the new approach that emerged from these researches. The following is the revised paragraph. "Research demonstrates that the variation in object categorization across the visual field is attributable to the inherently inhomogeneous nature of high-level visual representations. Although current convolutional neural networks impose homogeneous category representations across the visual field, existing brain-based evidence emphasizes that category-selective regions in the high-level visual cortex do not evenly sample the visual field (Kay et al., 2015; Le et al., 2017; Poltoratski et al., 2021; Silson et al., 2016). Our results align with this research, reinforcing the idea that high-level visual representations are inhomogeneous across visual space. This correspondence between behavioral observations and neural processing supports our understanding of visual perception and the organization of the visual cortex, highlighting the complexity of object recognition in various visual contexts." Minor comments:

Page 5. Participants: 'The test was conducted without any intervention in the process.' What do the authors mean by 'intervention'? The statement refers to our ethical commitments, ensuring that no interventions, such as electrical or pharmacological methods (e.g., drugs, TDCS, TACS, TMS), were used. This is part of our ethical guidelines, which emphasize that the test was conducted without any external manipulation affecting the process. We have revised the sentence as follows: "The test was conducted without any external interventions, such as electrical stimulation or pharmacological methods." Page 6. Data analysis: '...across all trails...' instead of trials.

We changed this sentence.

Page 9. The two sentences describing Figure S1 seem to repeat the same conclusion: 'There was similar upper/lower visual field bias for chair/body category in other levels of the visual signal.' 'The lower/upper field bias of body/chair detection was observed across all levels of the visual signal (Table S2).' Thanks. We omitted the repeated sentences.

Page 12. 'This location dependent object recognition seems to emerge during early stages of development and depends on the statistics of sensory experience for each specific stimulus across the visual field (Tsurumi et al., 2021)'.

The reference provided (Tsurumi et al., 2021) is a Vision Sciences Society Annual Meeting Abstract, however, a paper following this abstract has already been published: Tsurumi et al.,2023 (Development of upper visual field bias for faces in infants. Developmental Science).

We have replaced this reference by published article.

Missing space before parenthesis:

Page 14. '...homogeneity of representation across the visual field(Stewart et al., 2020)'.

Page 15. '...manifest itself in local bias(Legge et al., 2001b)'.

Thanks. We modified them.

References: some references appear twice, for example: Barbot A, Xue S, Carrasco M (2021a); Himmelberg MM, Winawer J, Carrasco M (2020a); Cannon MW (1985a); Legge GE, Mansfield JS, Chung STL (2001a); Quek GL, Finkbeiner M (2016a); Thomas NA, Nicholls MER (2018a).

Thank you for your attention. we have modified the references section and removed the duplicate references.
